# Evaluation of the Prevalence of White Spot Lesions During Fixed Orthodontic Treatment Among Patients Reporting for Correction of Malocclusion: A Prevalence Study

**DOI:** 10.7759/cureus.42134

**Published:** 2023-07-19

**Authors:** Awanindra Kumar Jha, Subhash Chandra, Daya Shankar, Dhyan Chand Murmu, Mohammad Kashif Noorani, Nishant Kumar Tewari

**Affiliations:** 1 Department of Orthodontics and Dentofacial Orthopaedics, Rajendra Institute of Medical Sciences, Dental Institute, Ranchi, IND; 2 Department of Dentistry, Patna Medical College and Hospital, Patna, IND; 3 Department of Dentistry, Rairangpur Government Hospital, Rairangpur, IND; 4 Department of Prosthodontics, Om Sai Dental & Cosmetic Centre, Patna, IND; 5 Department of Oral and Maxillofacial Surgery, Kiran Poly Clinic, Patna, IND

**Keywords:** dental hygiene, demineralization, fixed orthodontic patients, white spot lesions, prevalence

## Abstract

Background: Despite recent breakthroughs in caries preventive measures, one of the biggest issues clinicians confront is preventing demineralization while undergoing orthodontic therapy. The buildup of plaques around orthodontic brackets over time causes white spot lesions (WSLs). The goal of the present research was to assess the prevalence of WSLs in patients undergoing orthodontic treatment before starting therapy and at six and 12 months into therapy, adopting the visual examination approach.

Materials and methods: We looked for WSLs on tooth surfaces gingival to an archwire because this is the area most likely to experience enamel demineralization during orthodontic treatment. The visual assessment was conducted using the following scale at baseline, six months, and 12 months for orthodontic patients: score 0: no demineralization or noticeable white patches on the surface; score 1: mild demineralization with a visible white spot but no surface disruption; score 2: moderate demineralization with a noticeable WSL that has a roughened surface but does not need repair; and score 3: severe demineralization with a noticeable WSL that needs repair. Fisher's exact test was used after a chi-square analysis to determine whether there were any differences between all three categories (six months, 12 months, and control).

Results: The frequency of WSL in patients at 12 months of orthodontic treatment was 46.57%, while it was 11.86% in patients who just started orthodontic treatment. The difference was statistically significant (p = 0.01), showing that the frequency was greater in patients at 12 months of orthodontic treatment as compared to patients who had just started undergoing orthodontic treatment. The frequency of WSL in patients at six months of orthodontic treatment was 37.34%, while it was 11.86% in patients who just started orthodontic treatment. The difference was statistically significant (p = 0.03), showing that the frequency was greater in patients at six months of orthodontic treatment as compared to patients who had just started undergoing orthodontic treatment. The frequency of WSL in patients at six months of orthodontic treatment was 37.34%, while it was 46.57% in patients at 12 months of orthodontic treatment. The frequency was greater in patients at 12 months of orthodontic treatment as compared to patients at six months of orthodontic treatment; however, the difference was non-significant statistically (p = 0.76).

Conclusion: This clinical investigation revealed that the number of WSLs increased significantly during the first six months of treatment and then increased gradually until the final 12 months. During the first few months of treatment, doctors should assess the patients' dental hygiene habits and, if necessary, take further precautions to prevent demineralization.

## Introduction

When dental hygiene is neglected, there is a considerable risk of demineralization of the enamel during orthodontic procedures. Despite recent breakthroughs in caries preventive measures, one of the biggest issues clinicians confront is preventing demineralization practices while undergoing orthodontic therapy. The buildup of plaques around orthodontic brackets over time causes white spot lesions (WSLs) [1-5]. In addition to making routine oral hygiene practices more challenging, fixed orthodontic equipment significantly increases the stagnation areas for plaques that are typically less prone to caries formation [6].

The bacterial ecology of plaques rapidly changes after the placement of orthodontic fixed devices in the oral cavity. The plaque contains higher concentrations of acidogenic microorganisms, such as bacteria like Streptococcus mutans and Lactobacilli [7]. In individuals undergoing orthodontic treatment, high bacterial counts can cause plaque pH to decrease more rapidly as compared to non-orthodontic individuals [8]. As a result, caries develop more quickly in patients who have complete orthodontic apparatus. While the development of ordinary caries usually requires at least six months, WSLs can start to show up around the brackets as soon as one month after bracket installation. Oral biofilms are highly organized bacterial colonies in a hydrated extracellular polymeric substance (EPS) matrix. Oral biofilms develop in the same order, but their structural organization, composition, and colonizing bacteria may vary. The oral biofilm structure and bacterial viability have been quantified using a confocal laser scanning microscope (CLSM).

However, the reason behind mature biofilm bacteria's resistance to antibacterial therapies is unknown. Understanding biofilm structure is essential to understanding its specific characteristics and monitoring physical or chemical eradication methods. Oral biofilm bacteria resist more antimicrobials than planktonic bacteria. Understanding how adhesion affects oral biofilm production may help design efficient control strategies, as bacterial endurance and pathogenicity have often been linked to biofilm formation [9,10]. These kinds of lesions are frequently encountered, particularly in the gingival area, on the buccal faces of the teeth near brackets [1,5].

Although WSLs can appear in under a month, the development of these sorts of defects and their frequency at various stages of orthodontic therapy have not been studied. The ability to manage demineralization activity and stop lesions from progressing would be made possible through the early diagnosis of WSLs throughout orthodontic therapy [11,12]. Therefore, the goal of the present research was to assess the incidence of WSLs in patients undergoing orthodontic treatment before starting therapy and at six and 12 months into therapy, adopting the visual examination approach.

## Materials and methods

Before the start of the clinical study, ethical approval was taken from the institutional ethics committee of Rajendra Institute of Medical Sciences, Ranchi, India, with approval no. 279IEC/RIMS, dated June 23, 2021. Patients receiving treatments with fixed appliance orthodontics who were 12 years of age or older and volunteered to take part in the research were included as the study participants. Patients who have fluorosis lesions and yellow or brown stains and all patients who were advised for fluoride mouth rinse as a fluoride supplement were excluded from the study.

The sample size was calculated using the following formula: 

*n = (Z^2^ x p x q) / E^2^*,

where *n* is the required sample size, *Z* is the Z-score corresponding to the desired confidence level (e.g., 1.96 for a 95% confidence level), *p *is the estimated proportion of the expected prevalence of an outcome, *q* is 1 - *p*, and *E* is the desired margin of error or precision. 

The minimum sample size calculated was 50. White spots due to fluorosis teeth and undiagnosed white spots in early incipient caries may be confounding factors.

In this study, three categories of study participants were evaluated: Category 1: Study participants were evaluated for WSLs after 12 months of fixed orthodontic treatment. Category 2: Study participants were evaluated for WSLs after six months of fixed orthodontic treatment. Category 3: Study participants were evaluated for WSLs immediately after the beginning of orthodontic treatment. Category 3 acts as a control group and provides baseline data for the study.

Before starting orthodontic therapy, all the patients were advised to have professional oral prophylaxis. Brackets were bonded with Enlight Light Cure adhesive composite (Ormco Corporation, USA) recommended for orthodontic bonding. At the beginning of each week, a research assistant looked through the Department of Orthodontics calendar to find patients who qualified. Afterward, subjects who had already been identified were asked if they would take part in the study, and their informed consent was obtained. The same practitioner, who was blind to the patient's expected time for orthodontic therapy, took measurements on each participant in the trial. The accessibility of information that might otherwise reveal the length of prior treatment was minimized by the doctor by simply assessing individuals after the orthodontist eliminated wires and auxiliary attachments. The research assistant added the name of the patient's group after taking these measurements to the examination form.

On a clinical form, the treatment group area was left blank, and the patient's age, race, and gender were noted, along with the results of the visual examination done on the dental chair with good illumination. Before measurements were obtained, the archwire was removed, and cotton rolls were used to isolate the upper teeth from the right maxillary premolar to the left maxillary premolar and air-dry them for five seconds. WSLs were measured by the same clinician who is blinded for the duration of the orthodontic treatment. The gingival tooth surface around the bracket was examined under a dental chair light by the same clinician.

We only looked for WSLs on tooth surfaces from the gingival to the archwire because this is the area most likely to experience enamel demineralization during orthodontic treatment. The visual assessment was conducted using the following scale: score 0: no demineralization or noticeable white patches on the surface; score 1: mild demineralization with a visible white spot but no surface disruption; score 2: moderate demineralization with a noticeable WSL that has a roughened surface but does not need repair; and score 3: severe demineralization with a noticeable WSL that needs repair [13]. After the completion of the study, patients with Grade 1 and Grade 2 WSL were advised for 0.2% sodium fluoride mouth rinse. Patients with Grade 3 WSL were advised restoration with fluoride-releasing glass ionomer cement.

Statistical analysis

Fisher's exact test was used after the chi-square analysis to determine whether there were any differences between all the three categories (six months, 12 months, and control) in the frequency of displaying at least one WSL. The logistic regression method was applied to assess the numerous impacts of the group (duration in treatment) and gender and to identify how groups and gender interacted. By utilizing analysis of variance, variation in the mean quantity of white spots among the groups was examined. With logistic regression, the incidence of white spots per tooth type was assessed. The confidence interval was set at 95%. The cut-off for significance was chosen at p = 0.05.

## Results

The mean age of the study participants in categories one, two, and three was 18.5± 1.3, 18.2± 1.4, and 17.2± 1.1 years old, respectively.

The difference was statistically significant (p = 0.01), showing that the frequency was greater in patients at 12 months of orthodontic treatment as compared to patients who had just started undergoing orthodontic treatment. The difference was statistically significant (p = 0.03), showing that the frequency was greater in patients at six months of orthodontic treatment as compared to patients who had just started undergoing orthodontic treatment. The frequency was greater in patients at 12 months of orthodontic treatment as compared to patients at six months of orthodontic treatment; however, the difference was non-significant statistically (p = 0.76) (Table 1).

**Table 1:** Frequency of study participants with WSLs WSLs: white spot lesions; n: number of patients; %: percentage

Categories	12 months	6 months	Control
No WSL, n (%)	39 (53.42)	47 (62.67)	52 (88.14)
WSLs present, n (%)	34 (46.57)	28 (37.34)	7 (11.86)

The mean WSL per patient was greater in patients at 12 months of orthodontic treatment than in patients at the beginning of orthodontic treatment. The difference was significant statistically (p = 0.001). The mean WSL per patient was greater in patients at six months of orthodontic treatment than in patients at the beginning of orthodontic treatment. The difference was significant statistically (p = 0.03). The mean WSL/patient was greater in patients at 12 months of orthodontic treatment than in patients at six months of orthodontic treatment. The difference was non-significant statistically (p = 0.43).

The percentage of patients with one to three WSLs was greater in patients at 12 months of orthodontic treatment than in patients at the beginning of orthodontic treatment. The difference was significant statistically (p = 0.021). The percentage of patients with one to three WSL was greater in patients at six months of orthodontic treatment than in patients at the beginning of orthodontic treatment. The difference was significant statistically (p = 0.002). The percentage of patients with one to three WSLs was greater in patients at 12 months of orthodontic treatment than in patients at six months of orthodontic treatment. The difference was non-significant statistically (p = 0.65).

The percentage of patients with four or more WSL was greater in patients at 12 months of orthodontic treatment than in patients at the beginning of orthodontic treatment. The difference was significant statistically (p = 0.04). The percentage of patients with four or more WSLs was greater in patients at six months of orthodontic treatment than in patients at the beginning of orthodontic treatment. The difference was significant statistically (p = 0.018). The percentage of patients with four or more WSLs was greater in patients at 12 months of orthodontic treatment than in patients at six months of orthodontic treatment. The difference was non-significant statistically (p = 0.96) (Table 2).

**Table 2:** Distribution of WSLs per study participant WSLs: white spot lesions; n: number of patients; %: percentage

Category	12 months	6 months	Control
Mean WSLs/patient (±SD)	1.24± 0.33	0.96 ±0.33	0.25±0.35
No WSLs, n (%)	39 (53.42)	46 (63.01)	52 (88.13)
1 to 3 WSLs, n (%)	25 (34.24)	16 (21.91)	7 (11.86)
≥4 WSLs, n (%)	9 (12.32)	11 (15.06)	0 (0)

The percentage of male study participants who were evaluated for WSL at 12 months of orthodontic treatment that had WSLs was 75.75%. The percentage of female study participants who were evaluated for WSL at 12 months of orthodontic treatment that had WSLs was 22%. The percentage of male study participants who were evaluated for WSL at six months of orthodontic treatment that had WSLs was 69.99%. The percentage of female study participants who were evaluated for WSL at six months of orthodontic treatment that had WSLs was 15%. The percentage of male study participants who were evaluated for WSL immediately after the beginning of the orthodontic treatment that had WSLs was 12.13%. The percentage of female study participants who were evaluated for WSL immediately after the beginning of the orthodontic treatment that had WSLs was 2.5% (Table 3).

**Table 3:** Effect of gender on WSL development WSL: white spot lesion; %: percentage

Category	12 months	6 months	Control
Number (%) of males with WSLs	25 (75.75)	23 (69.99)	4 (12.13)
Number (%) of females with WSLs	9s (22)	06 (15.00)	1 (2.5)

## Discussion

The goal of the present research was to assess the prevalence of WSLs in patients undergoing orthodontic treatment before starting therapy and at six and 12 months into therapy, adopting the visual examination approach. The frequency of WSLs in patients at 12 months of orthodontic treatment was 46.57%, while it was 11.86% in patients who just started orthodontic treatment. The difference was statistically significant (p = 0.01), showing that the frequency was greater in patients at 12 months of orthodontic treatment as compared to patients who had just started undergoing orthodontic treatment. The area-wise prevalence of WSLs as extracted from various studies is mentioned in Table 4.

**Table 4:** Percentage prevalence of WSLs based on area WSL: white spot lesion

Country/area	Study design	Prevalence of WSL (%)
United States	Cross-sectional	15.2
Brazil	Cohort	28.6
Australia	Case-control	10.8
United Kingdom	Cross-sectional	12.1
Japan	Longitudinal	8.3

The frequency of WSLs in patients at six months of orthodontic treatment was 37.34%, while it was 11.86% in patients who just started orthodontic treatment. The difference was statistically significant (p = 0.03), showing that the frequency was greater in patients at six months of orthodontic treatment as compared to patients who had just started undergoing orthodontic treatment. The frequency of WSLs in patients at six months of orthodontic treatment was 37.34%, while it was 46.57% in patients at 12 months of orthodontic treatment. The frequency was greater in patients at 12 months of orthodontic treatment as compared to patients at six months of orthodontic treatment; however, the difference was non-significant statistically (p = 0.76).

According to a previous study of the published literature on the occurrence of WSLs, the majority of pertinent research reported the existence of these lesions after orthodontic therapy was finished. The frequency of WSLs fluctuates based on the evaluation method. In their study employing a visual inspection approach, Gorelick et al. [1] found that 50% of patients continued to have solitary or multiple WSLs after therapy. At the conclusion of orthodontic therapy, Boersma et al. [11] used quantified light fluoroscopy to assess the frequency of WSLs and found that 97% of patients exhibited either solitary or multiple lesions. These investigations suggest that mineral loss is a substantial clinical issue that causes an unfavorable aesthetic presentation and, in some extreme circumstances, may call for restorative therapy.

The mean WSL per patient was greater in patients at 12 months of orthodontic treatment than in patients at the beginning of orthodontic treatment. The difference was significant statistically (p = 0.001). The mean WSL per patient was greater in patients at six months of orthodontic treatment than in patients at the beginning of orthodontic treatment. The difference was significant statistically (p = 0.03). The mean WSL per patient was greater in patients at 12 months of orthodontic treatment than in patients at six months of orthodontic treatment. The difference was non-significant statistically (p = 0.43). The percentage of patients with one to three WSLs was greater in patients at 12 months of orthodontic treatment than in patients at the beginning of orthodontic treatment. The difference was significant statistically (p = 0.021). The percentage of patients with one to three WSLs was greater in patients at six months of orthodontic treatment than in patients at the beginning of orthodontic treatment. The difference was significant statistically (p = 0.002). The percentage of patients with one to three WSLs was greater in patients at 12 months of orthodontic treatment than in patients at six months of orthodontic treatment. The difference was non-significant statistically (p = 0.65).

There is a significant danger of demineralization of the enamel after orthodontic operations when dental care is neglected. Despite recent advances in caries preventive strategies, one of the largest challenges orthodontists face is preventing demineralization when patients are receiving orthodontic treatment [13-15]. WSLs are brought on by the accumulation of plaques surrounding orthodontic brackets over time. Fixed orthodontic devices considerably increase the number of plaque persistence sites on the outside of the teeth, which are normally less susceptible to caries formation, in addition to making routine oral hygiene practices demineralization more difficult [16-18].

The percentage of patients with four or more WSLs was greater in patients at 12 months of orthodontic treatment than in patients at the beginning of orthodontic treatment. The difference was significant statistically (p = 0.04). The percentage of patients with four or more WSLs was greater in patients at six months of orthodontic treatment than in patients at the beginning of orthodontic treatment. The difference was significant statistically (p = 0.018). The percentage of patients with four or more WSL was greater in patients at 12 months of orthodontic treatment than in patients at six months of orthodontic treatment. The difference was non-significant statistically (p = 0.96) (Table 2).

When compared to people who are not receiving orthodontic treatment, elevated bacterial numbers can cause plaque pH to drop more quickly in orthodontic patients [19]. As a result, patients with fully functional orthodontic appliances experience caries development more quickly. While normal caries typically take at least six months to develop, WSLs can begin to appear around brackets as soon as one month following bracket placement. The buccal faces of the teeth close to braces are usually affected by these diseases, especially in the gingival region [20].

Oral multi-species biofilm-induced infections may be treated with antibiofilm host defense (antimicrobial) peptides. Antibiofilm peptides, which specifically target microbial biofilms, can both kill and disperse pre-existing biofilms generated by Gram-negative and Gram-positive bacteria. When mechanical teeth cleaning is impractical, difficult, or insufficient, chlorhexidine rinses should be used in the short and medium terms because they have been shown to be both safe and effective [21].

The present study has some limitations. First, the development of these kinds of defects and their frequency at various stages of orthodontic therapy have not been examined, despite the claim that WSLs can arise in less than a month. WSLs were evaluated on a limited number of participants in a limited population. The number of patients choosing fixed orthodontic treatment is less because it is an expensive procedure offered in tertiary medical facilities. Patients receiving fixed orthodontic treatment have been seen to have WSLs. For complex malocclusions, fixed orthodontic treatment is advanced and requires a skilled orthodontist. Early detection of WSLs during orthodontic therapy would enable control of the demineralization activity and halt the progression of lesions.

## Conclusions

Within the constraints of this study, it was discovered that WSLs among patients with fixed orthodontics constituted a cause for concern. WSL prevalence rates are quite high and significant among patients receiving orthodontic treatment. This clinical investigation revealed that the number of WSLs increased significantly during the first six months of treatment and then increased gradually until the final 12 months. Hence, additional strategies to reduce the risk of the development of these lesions need to be considered. During the first few months of treatment, orthodontists should assess the patients' dental hygiene habits and, if necessary, take further precautions to prevent demineralization.
